# Identification of genes associated with abiotic stress tolerance in sweetpotato using weighted gene co‐expression network analysis

**DOI:** 10.1002/pld3.532

**Published:** 2023-10-03

**Authors:** Mercy Kitavi, Dorcus C. Gemenet, Joshua C. Wood, John P. Hamilton, Shan Wu, Zhangjun Fei, Awais Khan, C. Robin Buell

**Affiliations:** ^1^ Research Technology Support Facility (RTSF) Michigan State University East Lansing Michigan USA; ^2^ Center for Applied Genetic Technologies University of Georgia Athens Georgia USA; ^3^ International Potato Center Lima Peru; ^4^ International Maize and Wheat Improvement Center (CIMMYT), ICRAF House Nairobi Kenya; ^5^ Department of Crop & Soil Sciences University of Georgia Athens Georgia USA; ^6^ Boyce Thompson Institute Cornell University Ithaca New York USA; ^7^ Present address: Plant Pathology and Plant‐Microbe Biology Section, School of Integrative Plant Science Cornell University Geneva New York USA; ^8^ Institute of Plant Breeding, Genetics, & Genomics University of Georgia Athens Georgia USA

**Keywords:** abiotic stress, differentially expressed genes, sweetpotato, weighted gene co‐expression network

## Abstract

Sweetpotato, *Ipomoea batatas* (L.), a key food security crop, is negatively impacted by heat, drought, and salinity stress. The orange‐fleshed sweetpotato cultivar “Beauregard” was exposed to heat, salt, and drought treatments for 24 and 48 h to identify genes responding to each stress condition in leaves. Analysis revealed both common (35 up regulated, 259 down regulated genes in the three stress conditions) and unique sets of up regulated (1337 genes by drought, 516 genes by heat, and 97 genes by salt stress) and down regulated (2445 genes by drought, 678 genes by heat, and 204 genes by salt stress) differentially expressed genes (DEGs) suggesting common, yet stress‐specific transcriptional responses to these three abiotic stressors. Gene Ontology analysis of down regulated DEGs common to both heat and salt stress revealed enrichment of terms associated with “cell population proliferation” suggestive of an impact on the cell cycle by the two stress conditions. To identify shared and unique gene co‐expression networks under multiple abiotic stress conditions, weighted gene co‐expression network analysis was performed using gene expression profiles from heat, salt, and drought stress treated ‘Beauregard’ leaves yielding 18 co‐expression modules. One module was enriched for “response to water deprivation,” “response to abscisic acid,” and “nitrate transport” indicating synergetic crosstalk between nitrogen, water, and phytohormones with genes encoding osmotin, cell expansion, and cell wall modification proteins present as key hub genes in this drought‐associated module. This research lays the groundwork for exploring to a further degree, mechanisms for abiotic stress tolerance in sweetpotato.

## 1 INTRODUCTION

Crops are exposed to abiotic and biotic stress, which can lead to adverse effects on growth and productivity (Hussain et al., 2019). Changing climate conditions including extreme temperature and water deficit conditions have the potential to substantially limit crop growth and productivity (Dahal et al., 2019; Hussain et al., 2019; Zhou et al., 2017). These production limitations, coupled with the need to expand feed and food production to meet a growing world population, will most likely require expansion of production areas to marginal agricultural lands. Understanding how plants perceive abiotic stress signals and adapt to adverse environmental conditions can facilitate development of improved cultivars essential for global food security.

Sweetpotato, *Ipomoea batatas* (L.) Lam., which originated in Central America, is a widely cultivated crop with a world production in 2019 of 91.8 metric tons (FAOSTAT, 2021). Sweetpotato root flesh can be white, cream, yellow, orange, or purple in color and is an excellent source of vitamins (C, E, K and several B vitamins) and serves as a major caloric source for sub‐Saharan Africa (Bovell‐Benjamin et al., 2009; Low et al., 2017). However, only orange fleshed sweetpotato varieties are rich in beta‐carotene, which is converted into vitamin A, an essential vitamin for a strong immune system, healthy skin, as well as vision and eye health (Low & Thiele, 2020). Orange fleshed sweetpotato varieties (OFSP) are good source of nondigestible dietary fiber, minerals, vitamins, and antioxidants (Dako et al., 2016; Neela & Fanta, 2019). OFSPs have high β‐carotene levels but low dry matter content (18%–25%), and are typically sweet with a moist texture after cooking and are commercially popular in the United States of America (Grace et al., 2014; Islam et al., 2016; Liao et al., 2008). Orange‐fleshed sweetpotato is a low‐priced crop and can be a year‐round source of vitamin A in most of sub‐Saharan Africa and other places where sweetpotato is a staple food. Most orange‐fleshed sweetpotato varieties contain 3000–16,000 μg 100 g(−1) of β‐carotene and contribute to 250 to 1300 μg 100 g(−1) Retinol Activity Equivalents (RAE) (Dako et al., 2016; Olaniran et al., 2023). Using OFSP, vitamin A status can be improved as well as increase bioavailability of different micro‐nutrients such as Fe, Zn, Ca, and Mg, this has been reported to reduce vitamin A deficiency and child mortality rates by 23% to 30% (Gurmu et al., 2014; Hernández Suárez et al., 2016; Olaniran et al., 2023).

Currently, efforts to address vitamin A deficiency in sub‐Saharan Africa are focused on use of OFSP (Low et al., 2017). Beauregard, an orange‐fleshed cultivar with low dry matter (Lau et al., 2018; Rolston et al., 1987), has played a critical role in the improvement of African sweetpotato cultivars with increased β‐carotene via introgression of *Orange* (*Or)* alleles through breeding (Gemenet et al., 2020). Beauregard has been used in both direct and recurrent crosses for studying of agronomic traits such as sweetpotato root‐knot nematode resistance, drought tolerance and understanding of the relationship between starch and beta‐carotene in elite breeding lines (Cervantes‐Flores et al., 2011; Gemenet et al., 2020; Lau et al., 2018; Mollinari et al., 2020).

Plants have evolved interconnected regulatory pathways enabling response(s) to biotic (including various plant pathogens such as bacteria, fungi, viruses, nematodes, and insects) and abiotic (e.g., heat, salinity, drought, heavy metals, and cold) stresses. Understanding these mechanisms can aid in understanding and improving stress tolerance and the potential to recover yield under stress (Atkinson & Urwin, 2012; Bashir et al., 2019; Gull et al., 2019). Furthermore, transcriptional regulatory networks in *Arabidopsis thaliana* have revealed some of the underlying molecular, cellular, physiological, and biochemical bases of abiotic stress responses (Barah et al., 2016; Berens et al., 2019; Nakashima et al., 2009; Yamada et al., 2010). This includes physiological changes such as altering stomatal pore apertures thereby enabling optimized CO_2_ uptake while minimizing water loss (Nilson & Assmann, 2007) as well as minimizing oxidative damage from the production of reactive oxygen species (ROS) (Arbona et al., 2017).

Phytohormones are crucial integrators of the adaptive mechanisms of stress responses. Abscisic acid (ABA)‐dependent and ABA‐independent pathways have been described in the response to drought stress (Yamaguchi‐Shinozaki & Shinozaki, 2006). Plant leaves respond to water deficit by increasing ABA biosynthesis, transport and accumulation thereby triggering stomatal closure that decreases gas exchange rate, respiration, and photosynthetic activity (Osakabe et al., 2014; Yamaguchi‐Shinozaki & Shinozaki, 2006). ABA action not only targets guard cells and the induction of stomatal closure but also systemically signals to adapt to water limitations. Due to its induction by various stresses, ABA is considered a plant stress hormone (Swamy & Smith, 1999; Tuteja, 2007). Several transcription factors (TFs) are known to regulate the expression of abiotic stress‐responsive genes either via the ABA‐dependent or ABA‐independent pathway (Xiong et al., 2002).

Gene expression profiling experiments have provided insights to the basis of molecular responses to environmental stressors for numerous plants species including Arabidopsis (Zhao et al., 2019), barley (Baidyussen et al., 2020), maize (Frey et al., 2015; Hoopes et al., 2019), potato (Gong et al., 2015; Rensink et al., 2005), rice (González‐Schain et al., 2019, 2016; Mangrauthia et al., 2017; Tang et al., 2020), sweetpotato (Arisha, Ahmad, et al., 2020; Lau et al., 2018), wheat (Kumar et al., 2021), tobacco (Li et al., 2019), and desert poplar (Zhang et al., 2017). Access to large‐scale gene expression profiling datasets from a wide range of species under diverse abiotic stress conditions has revealed not only stress‐specific responses but also shared or common responses to multiple abiotic stress conditions. Weighted gene co‐expression network analysis (WGCNA) clusters genes into networks based on the connectivity of the gene expression (Langfelder & Horvath, 2008; Zhang & Horvath, 2005) in which genes with similar expression patterns are grouped into the same module (clusters of highly interconnected genes) suggestive of similar functions and/or potentially common biological regulatory roles (Zhou, Huang, et al., 2018). This method has been successfully used to identify modules associated with biological processes in different plant species (Du et al., 2017; Greenham et al., 2017; Hoopes et al., 2019; Shaik & Ramakrishna, 2013; Wang et al., 2020). Within these modules are hub nodes (genes) which are highly connected, central to the network's architecture, and are hypothesized to have an influential role in regulating network structure (Albert et al., 2000; Barabási & Oltvai, 2004). Gene connectivity has been used for identifying hubs as well as identifying differentially connected genes (Arnatkeviciute et al., 2021; Liu, Gu, et al., 2019; Panahi & Hejazi, 2021).

Generation of the reference genome sequence of the wild diploid sweetpotato relative, *Ipomoea trifida* (Kunth) G. Don (Wu et al., 2018), has permitted transcriptome analysis of cultivated hexaploid sweetpotato (Bednarek et al., 2021; Gemenet et al., 2020; Suematsu et al., 2020). With respect to abiotic stress, transcriptomic responses of sweetpotato under simulated drought conditions (Arisha, Ahmad, et al., 2020; Lau et al., 2018), heat stress (Arisha, Aboelnasr, et al., 2020), and salt stress (Luo et al., 2017; Meng et al., 2020; Yang, Zhu, et al., 2020) have been reported. Here, we examined gene‐expression patterns following heat and salt stress treatments in leaves of the OFSP cultivar Beauregard and identified shared and stress‐specific differential gene expression over a 48‐h time course. A broader view of abiotic stress in cultivated sweetpotato was established by constructing gene co‐expression networks using gene expression datasets from this study combined with a previously published simulated drought stress dataset (Lau et al., 2018).

## 2 MATERIALS AND METHODS

### 2.1 Experimental design and stress treatments

Empty glass culture tubes (25 mm × 150 mm) with clear polypropylene lids (https://microclone.com/) were sterilized in the autoclave at 121°C for 20 min at 15 psi before filling with 15‐mL MPB solid medium (Murashige and Skoog salts, 3% sucrose, 2‐mg/L calcium pantothenate, 100‐mg/L l‐arginine, 200‐mg/L ascorbic acid, 20‐mg/L putrescine‐HCl, 10‐mg/L GA_3_, .3% Phytagel, pH 5.7) and sterilizing again. The autoclaved media was allowed to cool in a sterile environment after which it was ready for use. Actively growing sterile in vitro shoots were used as explants instead of greenhouse plants due to their high response to shoot regeneration and proliferation and axenic nature. In a laminar airflow cabinet bench (Purair® FLOW™ Series laminar flow; https://www.labdepotinc.com/), explants were obtained from in vitro plantlets of sweetpotato cultivar “Beauregard” (CIP440132) from the International Potato Center (CIP) Genebank. Young and healthy shoots (~3.0 mm long), with axillary buds (two nodes) were excised and collected using sterilized scalpel and forceps. In each test tube, a single cutting (with no less than two nodes) was inserted into the culture keeping the position of the nodes upside. The shoot cultures were kept in a growth room at 28°C/20°C day/night temperature with 12‐h light and 12‐h dark conditions, under 3000 lx and relative humidity of 70% for 14 days to allow for shoot elongation. The plants (with leaves and roots) were then transferred to sterile MPB liquid medium (without agar) in a magenta jar, three shootings per box, and left to acclimatize for 7 days, after which either stress or control treatments were imposed. All experiments were carried out independently each with three replicates, each replicate containing three plants; the drought simulated experiments using polyethylene glycol (PEG) were previously reported by Lau et al. (2018). All treatment conditions were separately but concurrently applied with combined controls of salt and PEG (PEG_NaCl CTR). Salt stress was induced by adding 150‐mM NaCl to the medium while drought stress was simulated by creating osmotic pressure using PEG as described by Lau et al. (2018), treated plants were placed in the growth room at 28°C/20°C day/night. Control plantlets for salt and PEG were placed in fresh liquid medium, but no stress treatment was applied and placed in the growth room at 28°C/20°C day/night temperature. Heat stress was instigated by placing Beauregard in vitro plantlets at 40°C/32°C day/night in a controlled environment plant growth chamber while control plantlets were left at the optimal sweetpotato growth temperature of 28°C/20°C day/night. Leaf samples were collected at 24 and 48 h after stress (HAS) from heat, salt and PEG treated plants for stress, and no stress treated plants for controls.

### 2.2 Library construction and RNA sequencing

Leaf samples were collected in three biological replicates per treatment and time point. The TRIzol (Invitrogen) method was used to extract RNA and sequencing libraries were constructed using the protocol described in Zhong et al. (2011); sequencing performed on an Illumina HiSeq 2500 platform. Raw RNA‐Seq reads were filtered and trimmed using Cutadapt v2.10 (Martin, 2011) to remove adapter sequence, low‐quality bases (quality score <30), retaining only reads with a minimum read length of 70 nt. Libraries were sequenced to 151 nt and clipped to 101 nt using Cutadapt v2.10 (Martin, 2011) to equalize read length. Filtered clean reads were assessed for quality using FastQC v0.11.8 (https://www.bioinformatics.babra-ham.ac.uk/projects/fastqc) and viewed with MultiQC v1.9 (Ewels et al., 2016). Cleaned reads were aligned to the diploid reference genome *I. trifida* (NSP306; v3; http://sweetpotato.uga.edu/) (Wu et al., 2018) using HISAT2 v2.2.1 (Kim et al., 2019) with the options: ‐‐phred33 ‐‐min‐intronlen 20 ‐‐max‐intronlen 5000 ‐‐rna‐strandness R ‐‐dta‐cufflinks. Aligned files were sorted and merged using the samtools sort and merge options, respectively, in SAMTools v1.10 (Li et al., 2009). RNA‐seq data from Beauregard plants exposed to PEG to mimic drought were obtained from Lau et al. (2018).

Expression abundances (fragments per kilobase of exon model per million mapped fragments; FPKM) for each gene model were determined based on the RNA‐Seq read alignments using Cufflinks v2.2.2 (Trapnell et al., 2010) with parameters set to accurately weight reads mapping to multiple locations (‐‐multi‐read‐correct), minimum and maximum intron size (‐‐min‐intron‐length and ‐‐max‐intron‐length) of 10 and 5000 respectively in single end mode (‐‐library‐type fr‐first strand). Read counts were determined using HTSeq v0.12.3 (Anders et al., 2014) with the parameters ‐‐stranded = reverse ‐‐minaqual = 10 ‐type = exon ‐‐mode = union. For sample‐level quality controls, the relationship between the biological replicates was visualized using Pearson Correlation Coefficients (PCC) of gene expression abundances. Some libraries were sequenced more than once (technical replicates) and PCCs were checked prior to merging technical replicates; all technical replicates had a PCC > .97. To assess batch effects of the experiments, the regularized‐logarithm transformation (rlog) of expression count data was used to generate a Principal Component Analysis (PCA) and heat map in R v4.1 (https://cran.r-project.org/).

### 2.3 Differential gene expression

Using the unique combination of treatment and time‐point as a single factor, gene counts were used to detect differentially expressed genes (DEGs) with DESeq2 v1.28.1 (Love et al., 2014) between stress treatments and their respective controls in R v4.1. To detect genes that responded differentially to each treatment, contrasts were made between treatment and control at each time point using the model, design = ~treatment + time performed in DESeq2. Genes were filtered at an adjusted *p*‐value (padj) cut‐off < .05 and log_2_FoldChange (LFC) of 2.0. For visualization, “lfcShrink” function was used to account for low expression levels and MA plots were generated for each treatment and time point. Genes with a fold‐change between 2.0 for up regulated and −2.0 for down regulated genes with an adjusted *p*‐value cutoff of .05 were deemed DEGs. Gene Ontology (GO) enrichment tests were performed using topGO v2.50.0 in R v4.1 (Alexa & Rahnenfuhrer, 2021). *P*‐values < .05 from Fisher's Exact Tests using the weighted model was used as the test for significance (Benjamini & Hochberg, 1995).

### 2.4 Clustering and construction of gene co‐expression networks

Differentially expressed genes were clustered using the unsupervised K‐means clustering method with parameters: max_itr = 10,000 and n_clust = 25 in R (Oyelade et al., 2016). Co‐expression of genes was further explored using WGCNA in R v4.1.1 (Langfelder & Horvath, 2008). The expression abundances (FPKMs) of all gene isoforms (44,158) were used and filtered to 14,138 genes by removing all features whose counts were consistently low with a count of less than 5 in all the samples, (*x* < 5) in heat, salt, and drought treated samples.

Samples were initially clustered using the FlashClust tool to analyze sample height and detect and remove outliers. A soft thresholding power of 20 was chosen for subsequent co‐expression module construction following application of the scale‐free topology criterion (Barabási, 2009; Ruiz Vargas et al., 2014). Based on the topological overlap‐based dissimilarity measure (Gysi et al., 2018; Zhang & Horvath, 2005), genes were hierarchically clustered, and a dendrogram was used for module detection using the dynamic tree cut method (mergeCutHeight = .9, minModuleSize = 35). Modules were identified as gene sets with high topology overlap measure (TOM) generated by the adjacency and correlation matrices of gene expression profiles. Genes that did not fit in any modules were discarded from further analyses (gray module). Gene ontology enrichment of module genes was performed using topGO package; a *p‐*value or FDR < .05 was used to determine the significant enrichment (Alexa & Rahnenfuhrer, 2021).

Gene connectivity based on the edge weight (ranging from 0 to 1) was determined by TOM; the weights across all edges of a node were summed and used to define the level of connectivity, and nodes with high connectivity were considered hub genes. Intramodular connectivity measure, *kWithin*, or connectivity of a particular gene to all other genes within its same module was used to define hub genes in each module. *kTotal* was used to measure the connectivity of a gene to all other genes regardless of module (*kOut* is kTotal‐kWithin, and *kDiff* is kWithin‐kOut) (Rhead et al., 2020).

### 2.5 Gene orthology

OrthoFinder v2.5.4 (Emms & Kelly, 2015) was run with the longest peptide isoform of each *I. trifida* predicted peptide (http://sweetpotato.uga.edu/) with the *A. thaliana* TAIR10 (https://www.arabidopsis.org) and *Oryza sativa* v7 predicted proteome (Ouyang et al., 2007) obtained from Phytozome v12.1.5 (Goodstein et al., 2012). Transcription factors in *I. trifida* were obtained from iTAK (Zheng et al., 2016).

## 3 RESULTS AND DISCUSSION

### 3.1 Dynamic expression patterns of differentially expressed genes following heat and salt stress in cultivated sweetpotato

We used RNA‐seq to examine global transcriptional changes in sweetpotato leaves subjected to treatments of heat or NaCl (salt) stress and analyzed these along with a previously published RNA‐seq study in which PEG was used to mimic drought stress (Lau et al., 2018). A total of 70 RNA‐seq samples (technical and biological replicates) were processed revealing an overall alignment rate to the *I. trifida* reference genome between 72.12% and 85.2% (Table S1), consistent with previous analyses in which transcript reads from sweetpotato were aligned to *I. trifida*, a close diploid ancestor of the cultivated hexaploid *I. batatas* (Wu et al., 2018). Technical replicates were merged post‐alignment resulting in a total of 30 samples. Correlations between biological replicates were examined by generating pairwise PCCs for gene expression abundances (FPKMs); an average PCC value of .9985 and a minimum PCC of .9603 was observed (Table S2) suggesting high reproducibility and quality of the datasets.

**FIGURE 1:**
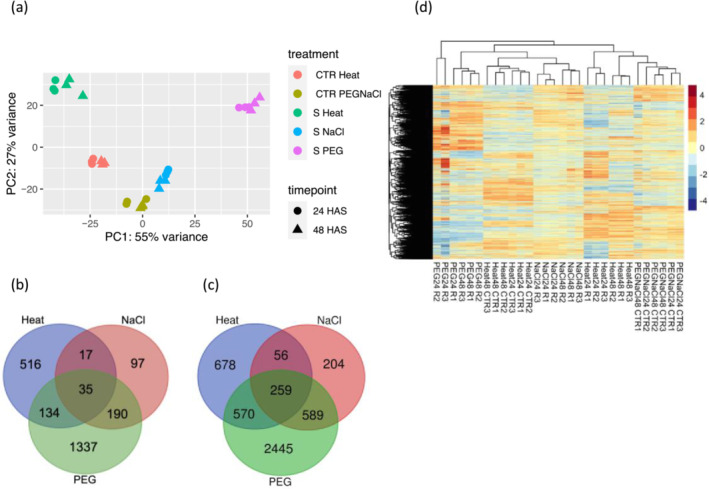
Assessment of inter‐ and intragroup variability. (a) Principal component analysis plot derived from 30,210 transcripts (nonZero counts) displaying all replicates along principal components 1 and 2 explaining 55% and 27%, respectively, of the variance in gene expression. Replicate samples from the same group cluster together, while samples from different groups form separate clusters indicating that the differences between groups are larger than those within groups; S and CTR denotes stress and control samples, respectively. Venn diagrams illustrate the number of differentially expressed genes (DEGs) as (b) up regulated and (c) down regulated in leaf tissue following heat or salt or PEG treatments of sweetpotato plants. The intersection shows genes commonly triggered by either two or three stress conditions. (d) Heatmap visualizing clustering expression patterns of 7127 DEGs up regulated (in red indicate highly expressed genes) or down regulated (in blue indicating low expression values, *p*‐value < .05) by salt (150‐mM NaCl) and heat (40°C) and drought stress treatment on sweetpotato plants suggesting stress specific and shared gene expression patterns under the three stress conditions. R and CTR denotes stress and control samples, respectively.

A principal component analysis (PCA; Figure 1a) was plotted to assess the relationships of the biological replicates and the extent of gene expression variation explained by the experimental variables. The PCA detected expression patterns regardless to significance or fold change thresholds and revealed a high similarity among the three biological replicates within each treatment. The tight clustering of biological replicates revealed that the largest variation in gene expression was due to the experimental conditions (stress vs. stress and stress vs. control) and minimal batch effects. The first principal component (PC1) explained 55% of the total variance (Figure 1a) separating PEG stress and heat stress samples. Limited separation of samples based on treatment time point was observed suggesting little time‐based effect on observed the transcriptional changes within the treatments.

Differences in gene expression responses to heat, salt and drought stress were observed in Beauregard leaves (Table S3). Contrast between the stress conditions versus their controls generated a set of DEGs that were up regulated or down regulated at each specific treatment and time point or both time points (24 and 48 HAS). Heat treatment triggered an up regulation of 433 genes at 24 HAS and 505 genes at 48 HAS (236 genes were up regulated in both time points). Under salt stress, 228 genes were up regulated at 24HAS, and 183 genes at 48HAS (72 genes were common between the time points). Imposing drought (PEG) treatment on Beauregard plants evoked up regulation of 2791 genes (1411 genes at 24 HAS, 1380 genes at 48 HAS; 1095 genes common to both time points).

Heat stress repressed 974 genes at 24 HAS and 1146 genes at 48 HAS (556 genes were common in both time points). Salt stress down regulated 955 genes and 426 genes at 24 HAS and 48 HAS respectively (272 genes were down regulated at both time points). There were more DEGs suppressed by PEG treatment, 3164 genes at 24 HAS and 2672 genes at 48 HAS while 1973 genes were common in both time points (Table S3). Lau et al. (2018) had reported a slightly higher number of up regulated and down regulated DEGs in PEG treated Beauregard leaves (up regulated; 2216 genes in each time point, while 3798 genes and 3285 genes were down regulated; genes in 24 HAS and 48 HAS subsequently). The discrepancy in the number of DEGs could have been due to the different log2fold‐change threshold (LFC 1.5) used in their analysis versus this analysis (LFC 2). The number of genes shared between the PEG treatment time points were 1094 genes up regulated and 1973 genes down regulated. We observed a decrease in gene expression in salt stress leaves with increased stress time (24 h vs. 48 h). This phenomenon was reported in salt sensitive rice cultivars and supports the hypothesis that roots of salt susceptible cultivars detect salinity stress by 24 h then signals appropriate responses to leaves, and then returned to basal levels, however genes response in tolerant leaves continued up to 72 h (Razzaque et al., 2019). This may perhaps be attributed to the maladaptive gene expression responses in the sensitive roots (Campbell‐Staton et al., 2021; Razzaque et al., 2019).

With the substantial overlap in genes differentially expressed by the stress conditions at the two time points, a three‐way Venn diagram was used to detect DEGs under at least two single stresses termed as common stress DEGs illustrating the DEGs unique to each stress treatment or shared sets of up regulated (Figure 1b) and down regulated (Figure 1c) genes between and among the treatments. The partitioning of DEGs unique to each stress is consistent with previous reports on the specificity of plant acclimations to individual stressors (Koch & Guillaume, 2020; Krasensky & Jonak, 2012) and visible in hierarchical clustering of DEGs (Figure 1d) in which shared as well as distinct clustering patterns are apparent. PEG and salt stress treatments had slightly higher number of shared up regulated (190 genes) and down regulated DEGs (589 genes) compared to PEG versus heat treatment (134 genes and 570 genes in up and down regulated categories respectively) or salt and heat treatments (52 genes up regulated and 315 genes down regulated). A high degree of similarity in several different ways, also in their molecular and genetic effects has been observed in drought and salinity stresses (Bartels & Sunkar, 2005; Dabab Nahas et al., 2019). These similarities include but are not limited to metabolic processes, such as increased levels of plant hormonal processes (e.g., ABA) or decrease in photosynthesis.

Genes that were triggered by the three treatments included the drought tolerance candidate gene *TAS14* homolog (itf03g07310) also mentioned by Lau et al. (2018) which was highly up regulated in PEG samples (24 h, LFC = 8.8009; *p*‐value 2.28E−82) with lower expressions in heat (LFC = 2.5516; *p*‐value; 2.60E−8) and salt (LFC = 2.6075, *p*‐value 1.43E−8) treated samples. The expression of TAS14, a LATE EMBRYOGENESIS ABUNDANT (LEA) protein of the dehydrin subfamily is known to be induced by salt, ABA or mannitol treatment (Muñoz‐Mayor et al., 2012). Some drought candidate genes such as the drought inducible DREB2A transcription factor (itf05g02470) was uniquely highly up regulated by >10 fold at 24 h (*p‐*value 6.40E−8) and 48 h (*p‐*value 1.99E−20) in PEG samples. DREB2A gene (AT2G40340, AT2G40350, AT1G75490), a member of the DREB subfamily A‐2 of ERF/AP2 transcription factor family is thought to be a key TF that functions under osmotic stresses in Arabidopsis (Sakuma et al., 2002, 2006). The ABC transporter gene homolog (itf01g08190) and 9‐*cis*‐epoxycarotenoid dioxygenase (NCED; itf01g21160), an ABA biosynthesis key enzyme, was up regulated by both PEG and NaCl treatments. ABC‐transporter knockouts have been found to cause sensitivity to drought stress (Kuromori et al., 2011). Furthermore, AtABCG21 and AtABCG22, which belong to the ABC transporter G family, were expressed specifically in guard cells and are involved in stomatal regulation; mutants resulted in increased water transpiration and drought susceptibility (Kuromori et al., 2017). Moreover, overexpression of the NCED gene using a stress‐inducible promoter enhanced drought resistance in petunia (Estrada‐Melo et al., 2015), grapevine (He et al., 2018), and rice (Changan et al., 2018).

Abiotic stressors such as drought, cold, salt, heat, oxidative stress, and heavy metal toxicity strongly influence photosynthesis (Cornic, 2000; Huang et al., 2019; Yang, Zhang, et al., 2020). Since drought response genes were extensively discussed by Lau et al. (2018), this analysis further compared the set of DEGs triggered by heat and NaCl treatments. The contrast between sweetpotato heat treated leaves versus control and NaCl treated leaves versus control resulted in differentially up regulation of 989 genes; 650 genes unique to heat stress, 287 genes unique to salt stress, and 52 genes common to both conditions (Tables [Link], [Link], [Link]). Inversely, 2357 genes were down regulated by heat and salt stress, while 1249 genes were unique to heat, 793 genes unique to salt stress, and 315 genes were down regulated in both stress treatments (Tables [Link], [Link], [Link]).

**FIGURE 2:**
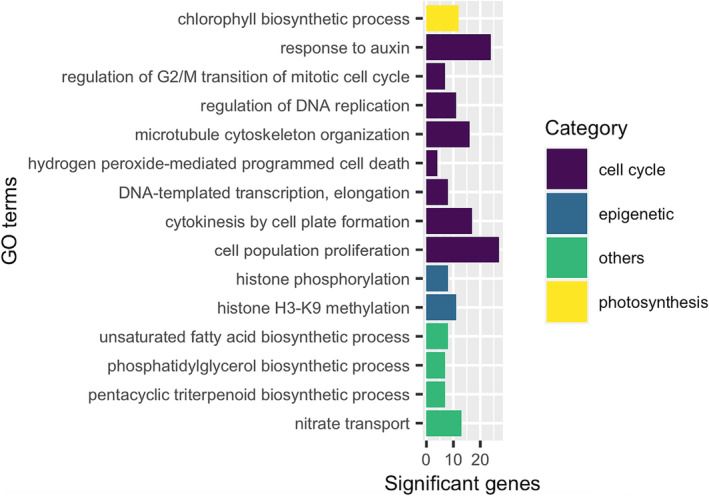
A graphical representation of the gene ontology terms under the biological processes category from the enrichment analysis on gene sets commonly down regulated (DEGs; LFC < − 2.0; *p‐*value < .05) by both heat stress and salt stress in Beauregard leaf tissue. The terms have been grouped (by color) based on the affected biological system.

Among the GO terms enriched in the common differentially down regulated genes overlapping between heat and salt stress was “chlorophyll biosynthetic process” (GO:0015995; *p‐*value .001) (Figure 2) with 12 photosynthetic genes including cycloartenol synthase (*CAS1*) which affects chlorophyll and carotenoid biosynthetic pigments in plants (Babiychuk et al., 2008; Luo et al., 2019). Various experiments have demonstrated a sharp decline in chlorophyll content (chlorosis) and a decrease in expression of some essential enzymes under stress (Allakhverdiev et al., 2008; Rossi et al., 2017). Destruction of the chloroplast ultrastructure by stress leads to a decrease in chlorophyll and ultimately lower photosynthetic activity (Hamani et al., 2020; Sidhu et al., 2017) explaining the down regulation of photosynthesis‐related genes (Figure 2). Moreover, the decrease in the chlorophyll content can also be caused by a decrease in the stomata aperture to limit water losses by evaporation and increased resistance to the entry of atmospheric CO_2_ necessary for photosynthesis. This is supported by the suppression of ribulose‐1,5‐bisphosphate carboxylase/oxygenase (Rubisco; ATCG00490; ortholog itf00g19690) gene, a lead enzyme in both photosynthetic carbon fixation and photorespiration at the 24 h of heat and salt stress time points (Roy & Cannon, 1988). Moreover, other genes such as the photosystem II reaction centers G (ATCG00430; ortholog; itf00g02070), B (itf00g02080), and D (itf02g22380) were repressed indicating a decrease in light absorption a mechanism to tolerate severe light and heat stress in plants (Yamamoto, 2016). In green plants, the chlorophyll pigment plays a critical roles in light energy harvesting during photosynthesis and charge separation in photosystem I (PSI) and photosystem II (PSII) in green plants (Luo et al., 2023).

Organ growth is divided into cell proliferation (increase in cell number) and the cell expansion stage (expansion to the final size). Arrest of the cell cycle in response to stress enables organisms to survive under fluctuating environmental conditions or DNA damage thereby allowing DNA repair to occur before DNA replication or mitosis resumes (Hu et al., 2016). DEGs that were down regulated by both heat and salt (Figure 2 and Table S9) were enriched in GO terms associated with “cell population proliferation” (GO:0008283392, *p‐*value 4.30E−13) and included genes encoding kinases and other genes previously reported to participate in plant cell cycle control in *A. thaliana* (Bertoni, 2018; Stals et al., 2000). During plant cytokinesis, Golgi/trans‐Golgi network‐derived vesicles are targeted to the plane of cell division where they fuse to form the cell plate. An ortholog of Qa‐SNARE (itf13g15140), a specialized syntaxin named KNOLLE (SYNTAXIN OF PLANTS 111, SYP111), is required for cytokinesis membrane fusion (Boutté et al., 2010; Lauber et al., 1997; Lukowitz et al., 1996; Touihri et al., 2011; Waizenegger et al., 2000) and exhibited significant down regulation following 48 h of heat (LFC = −6.8099, *p‐*value 4.54E−14) and 24‐h salt (LFC = −2.1419, *p‐*value 5.71E−5) stress (Table S9). Perturbation of cell cycle progression impacts the chromatin state and GO enrichment of terms associated with “histone phosphorylation” (GO:0016572; *p*‐value .001) and “histone H3‐K9 methylation” (GO:0051567; *p‐*value .013) were observed among the heat and salt common down regulated DEGs (Figure 2). Chromatin‐associated genes down regulated by heat and salt include two HISTONE 3.3 genes (itf09g16990: heat 48 h, LFC = −2.8211, *p‐*value 2.50E−6; salt 24 h, LFC = −2.0186, *p*‐value .0005 and itf06g11890: heat, 48 h, LFC = −2.8828, *p‐*value 7.21E−5; salt, 24 h, LFC = −2.4259, *p‐*value .0005) (Table S9). In addition, multiple Small Auxin Up regulated RNA (SAUR)‐like Auxin responsive protein family genes are among the 315 common down regulated DEGs under heat and salt stress (Table S9). SAUR‐like Auxin responsive protein family genes have been shown to regulate adaptive growth and are generally down regulated in response to abiotic stress.

Lipid components are important to biological systems and play critical roles in metabolic, regulatory, and structural domains during plant growth and development and environmental stresses adaptation (Hou et al., 2016). DEGs repressed by heat and salt stress were enriched in GO terms relative to biosynthetic processes including metabolism of unsaturated fatty acid, phosphatidylglycerol, and pentacyclic triterpenoid. Phosphatidylglycerin is one of the unique lipids of the lipid bilayer that plays a critical role in the function of photosystem II (PSII) in thylakoid membranes only lipid completely essential for oxygenic photosynthesis (Moellering & Benning, 2011). Recent reviews have shown a severe decrease of most glycerophospholipid species with polyunsaturated acyl chains as one of effects of long‐ and short‐term heat stress on the leaf lipidome following a heat shock without acclimation (Higashi & Saito, 2019). Corroborating our results, Qin, Lin, et al. (2020) observed a slight decrease of glycerolipids due to heat acclimation. Salt stress has also been reported to induce changes in membrane lipids of plants not only growing in maritime sands and salts marshes like the *Mesembryanthemum crystallinum* (Guo et al., 2022) but also in cultivated plants such as maize (Xu et al., 2021), *sorghum (*Ge et al., 2022), wheat, and alfafa (Li et al., 2023). The changes include the alterations of the lipid content, their fatty acid components, bilayer to non‐bilayer lipid ratio, and the activities of signaling lipids possibly regulating the membrane fluidity and permeability (Ge et al., 2022; Guo et al., 2022; Xu et al., 2021) A reduction in lipid content under salt stress was observed in salt sensitive barley compared to salt tolerant variety (Chalbi et al., 2013). Likewise, a significant decline in phosphatidylglycerin was seen in salt stressed *Sulla carnosa* and *Sulla coronaria* (Bejaoui et al., 2016).

Volatile terpenoids are specialized metabolites produced by plants as an adaptation to their environments including abiotic stress responses (Campbell et al., 2019; Murphy & Zerbe, 2020). A decrease in the expression levels of terpene synthase genes under increased heat and osmotic stresses was detected in roses (Yan et al., 2022), indicating the biosynthesis of terpenoids may be affected by abiotic stresses. The Arabidopsis gene AT3g45130 (LAS1, itf03g12580), and several cycloartenol synthase gene homologs (CAS1; itf03g22310, itf03g22340 and itf03g22370) which encode triterpene precursors were down regulated by both heat stress and NaCl stress (Table S9). Terpene synthase genes (CsTPS67, −69 and −71) were all suppressed and had inhibited expression in *Camellia sinensis* following salt and drought stress (Zhou et al., 2020). Furthermore, cycloartenol synthase genes were implicated in biosynthesis of unsaturated fatty acids (Table S10) revealing a possible co‐regulation of glycerol synthesis, terpene and fatty acid synthesis.

### 3.2 Heat stress in sweetpotato triggers a canonical heat shock response

**FIGURE 3:**
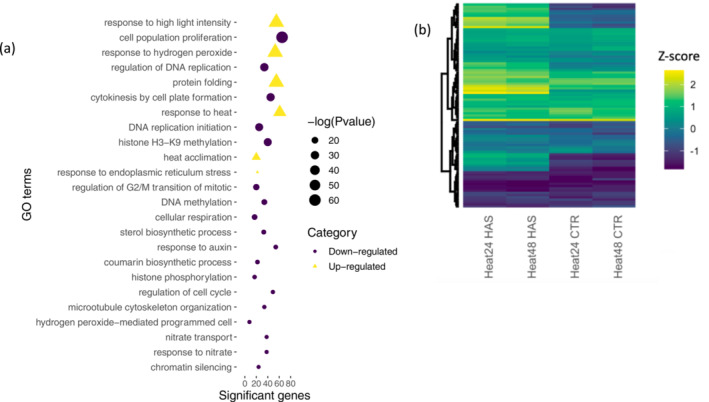
Effects of heat stress on Beauregard leaf tissues. (a) Biological process Gene Ontology (GO) terms enriched for the 702 up regulated and 1566 down regulated genes in response to heat stress in sweetpotato. (b) Heatmap of the expression (*Z*‐score) of heat shock proteins (HSPs) and heat shock transcription factors (HSFs) indicating their regulation (down or up regulated genes based on the scale color) in Beauregard leaves following heat stress; data generated from three biological replicates.

Differentially expressed genes following heat stress were significantly enriched in 115 GO terms in the three categories, *Biological process* (BP), *Cellular component* (CC), and *Molecular function* (MF) (*p*‐value ≤ .05; Table S10), across the two time points. Similar to that observed in Arabidopsis heat stress (Grinevich et al., 2019), heat‐related *Biological Process* GO terms such as “response to hydrogen peroxide” (GO:0042542: *p*‐value; 7.60E−28), “response to high light intensity” (GO:0009644; *p*‐value: 4.90E−27), “protein folding” (GO:0006457; *p*‐value: 1.70E−25), and “response to heat” (GO:0009408: *p*‐value; 4.60E−15) were significantly enriched (Figure 3a) among up regulated DEGs following heat stress suggesting that even brief exposure to 40°C resulted in perceived stress in *I. batatas* leaves.

Activation of transcriptional cascades following heat stress is common in many plant species. Following heat stress in Arabidopsis, 21 heat shock transcription factors (HSFs) and >2500 genes were differentially expressed (Busch et al., 2005). We identified 94 *I. trifida* orthologs that encode heat shock‐related proteins (HSPs) and HSFs (Table S11 and Figure 3b). Following heat stress, 28 HSP and 4 HSFs were differentially expressed with up regulation primarily occurring at 48 HAS rather than 24 HAS. A delay in the induction of heat shock proteins suggests that sweetpotato has a prolonged temperature threshold for the canonical heat shock response induction. This parallels the response in tepary bean relative to common bean, in which tepary bean which is adapted to hotter climates had a higher temperature threshold for induction of the canonical heat shock response (Moghaddam et al., 2021).

Arabidopsis chloroplast HSP mutants are sensitive to heat shock and exhibit variegated cotyledons, malformed leaves, growth retardation, and impaired root growth following heat stress (Lee & Schöffl, 1996; Schroda et al., 1999). In 48 h heat stressed Beauregard leaves, the chloroplast‐localized small heat shock protein HSP21 ortholog (itf00g29810, 48 h, Log_2_FC = 11.4101, *p*‐value 7.87E−20) was the highest and most significantly up regulated gene (Table S4), consistent with observations in Arabidopsis where HSP21 is involved in heat acclimation and was hyper‐induced in response to heat stress in Arabidopsis (Kim et al., 2014). CHLOROPLAST HEAT SHOCK PROTEIN 70–1 (itf04g21320, 48 h, Log_2_FC = 5.1012; *p*‐value 2.07E−117) was also significantly up regulated following heat stress (Table S4), yet the three paralogs of itf04g21320 [itf10g21850 (cpHSP 70‐1), itf01g02720 (cpHSP 70‐1), itf09g04300 (cpHSP 70‐2)] were not differentially expressed suggesting neo‐functionalization at the expression level in this gene family. Small heat stress proteins were the most abundant of the up regulated heat shock proteins in this study and are the most heat‐responsive in plants due to their dramatic induction and prevention of the aggregation of heat‐labile proteins that stabilize lipids at the plasma membrane while the HSP chaperones prevent and repair protein misfolding and aggregation to reduce cell damage (Guihur et al., 2021).

Heat shock transcription factors (HSF) play a critical role in the response to heat stress (Ohama et al., 2017) and HSFs have been identified in Arabidopsis (Nover et al., 2001), tomato, rice, maize (Scharf et al., 2012), wheat (Xue et al., 2014), apple (Giorno et al., 2012), poplar (Liu, Hu, & Zhang, 2019; Zhang et al., 2015), desert poplar (Zhang et al., 2016), pear (Qiao et al., 2015), tea (Liu et al., 2016), and grape (Liu, Chai, et al., 2018). In this study, HSFA6B gene (itf04g25720, 48 h, LFC = 3.3552, *p*‐value .0004, Table S4) was up regulated following heat stress. Simultaneous editing of *HSFA6a* and *HSFA6b* in Arabidopsis caused a reduction in reactive oxygen species (ROS) accumulation and increased expression of abiotic stress and ABA‐responsive genes, including those involved in ROS level regulation suggesting their involvement in abiotic stress tolerance through the regulation of ROS homeostasis in plants (Wenjing et al., 2020). HSFA2 genes are necessary for the maintenance of the acquired thermal tolerance (Lämke et al., 2016) and play a key role in the initiation of plant heat stress responses in Arabidopsis (Ohama et al., 2017). HSFA2 (itf12g20490, 48 h, LFC = 2.2994, *p*‐value 3.40E−5, Table S4) was up regulated upon heat stress. Increased HSFA2 levels in sweetpotato corroborate findings from Arabidopsis in which overexpression of the Arabidopsis *HsfA2* gene not only increased thermal tolerance but also salt/osmotic stress tolerance compared to the wild‐type (Ogawa et al., 2007).

Both enzymatic and nonenzymatic pathways cause H_2_O_2_ production in plants under natural and stressful conditions and is associated with plant development and growth. Heat stress induces membrane fluidity leading to accumulation of ROS and development of secondary stress responses under elevated temperature (Driedonks et al., 2015; Han et al., 2019; Mittler et al., 2012; Xiong et al., 2021). Although H_2_O_2_ is toxic at high concentrations, heat stress‐induced H_2_O_2_ is required for the effective expression of HSP genes in Arabidopsis (Liu, Zhang, et al., 2018; Volkov et al., 2006; Wang et al., 2014). HSFs are postulated to act as H_2_O_2_ sensors in the plants (Davletova et al., 2005; Hu et al., 2020; Miller & Mittler, 2006). Consistent with this hypothesis, 58 genes were enriched for the GO term “response to hydrogen peroxide” (GO:0042542, *p*‐value 7.60E−28) including the HEAT SHOCK TRANSCRIPTION FACTOR B2A; HSFB2A (itf13g03340; 24 h, LFC = 3.3064, *p*‐value 8.38E−11) following heat stress in sweetpotato.

We observed enrichment of GO terms in genes down regulated in response to heat stress associated with the cell cycle including “cell population proliferation” (GO:0008283, *p*‐value 3.20E−20), “cytokinesis by cell plate formation” (GO:0000911, *p*‐value 3.00E−09), “G2/M transition of mitotic” (GO:0010389, *p*‐value 1.90E−7), “DNA replication” (GO:0006275, *p*‐value 1.40E−11), and “DNA replication initiation” (GO:0006270, *p*‐value 5.50E−10) (Table S10). Increased cellular temperatures cause protein denaturation interrupting critical cellular processes and resulting in apoptosis and cell death (Gao et al., 2015; Gu et al., 2014; Matsuki et al., 2003). The cell cycle consists of G1, S, G2, and M stages with two major checkpoints at the G1/S checkpoint and G2/M. Cyclins in combination with cyclin‐dependent kinases (CDKs) drive the cell cycle. Abiotic stress induces signaling molecules that suppress the activities of CDKs via controlling the expression level of cyclins or regulating the posttranslational modification of CDKs therefore arresting or even exiting the cell cycle. Transient arrest of cell division at G1/S and G2/M transitions was reported in Arabidopsis following a short‐term (acute) exposure to moderate heat shock (Chao et al., 2017; Harashima et al., 2013; Kühl & Rensing, 2000; Nathans et al., 2021; Shimotohno et al., 2021; Takahashi et al., 2019), in tobacco BY2 cells under a mild heat stress (Jang et al., 2005), and in yeast and human cells (Leech et al., 2020). Moghaddam et al. (2021) observed down regulation of cellular process and cell cycle arrest following exposure to short moderate heat stress in heat tolerant tepary bean (*Phaseolus acutifolius*) but not heat sensitive common bean (*Phaseolus vulgaris*) suggesting adaptive responses to heat stress between these two *Phaseolus* species. In heat stressed sweetpotato, the cyclin‐dependent kinase B2;2 (CDKB2;2 itf07g23920, LFC = −2.17237, *p*‐value 1.24E−6) gene involved in cytokinesis and regulation of the G2/M transition of the mitotic cell cycle was down regulated.

### 3.3 Gene expression profiles in sweetpotato leaf tissue following salt stress

**FIGURE 4:**
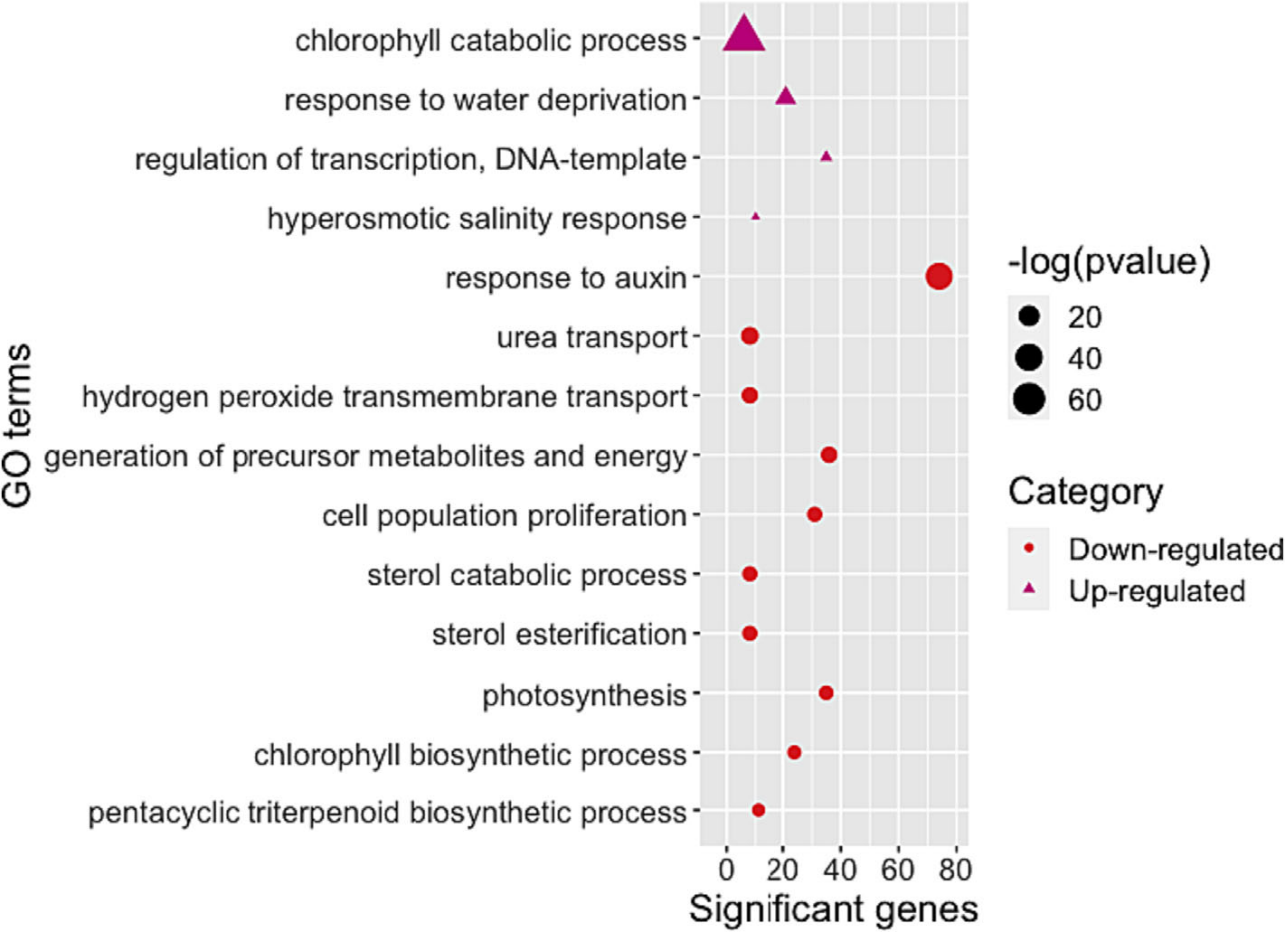
Effects of salt stress on Beauregard leaf tissues. Significant (*p* < .05) Biological Process Gene Ontology (GO) terms enriched for 339 up regulated (triangles) and 1108 down regulated (dots) differentially expressed genes in “Beauregard” leaf tissues at 24 and 48 h following salt stress.

High levels of salt lead to ion toxicity, hyperosmotic stress, and secondary stresses (Chakraborty et al., 2018). Na^+^, if accumulated in the cytoplasm, can be toxic to living cells adversely affecting K^+^ nutrition and vital plant physiological mechanisms including cytosolic enzymes, photosynthesis, and metabolism (Chakraborty et al., 2018; Shabala & Cuin, 2008). Salt stress impacts plant growth and development by reducing plant water potential, altering nutrient uptake, and increasing the accumulation of toxic ions (Shrivastava & Kumar, 2014). Among the enriched GO terms for genes differentially up regulated by salt stress were those associated with “response to water deprivation” (GO:0009414, *p*‐value 2.90E−5) and “hyperosmotic salinity response” (Figure 4 and Table S10). The homeobox gene (ATHB7, itf05g19720; LFC = 4.4975, *p*‐value 1.84E−20, 48 h), a putative TF that contains a homeodomain closely linked to a leucine zipper motif, was enriched in both GO terms. In Arabidopsis, ATHB7 and ATHB‐12 transcripts were found to be transcriptionally regulated in an ABA‐dependent manner and have been shown to mediate drought and respond to ionic osmotic stress (Olsson et al., 2004). Induction of NAC TF genes by drought, salt, and abscisic acid was observed in Arabidopsis (Sakuraba et al., 2015) and the NAC‐domain containing TF (itf05g25290: LFC = 2.3674, *p*‐value 2.81E−7) was up regulated during salt stress and enriched for response to water and hyperosmotic salinity. Other important genes enriched in salt stress samples were EID1 (itf07g18180: LFC = 2.8208, *p*‐value 1.69E−5), an F‐box protein involved in phytochrome A‐dependent light signal transduction, and fatty acid hydroxylase superfamily (itf04g03470: LFC = 2.8344, *p*‐value 1.36E−17), a wax biosynthesis‐related gene. It has been demonstrated that water deficit increases the amount of cuticular wax per unit area and leaf cuticle thickness in Arabidopsis plants to enhance their resistance (Lü et al., 2012).

Salt stress resulted in down regulation of genes associated with “chlorophyll biosynthetic process” (GO:0015995, *p*‐value 1.70E−6) and “photosynthesis” (GO:0015979, *p*‐value 9.00E−7) (Table S10). Accelerated leaf senescence induced by salt in detached mature sweetpotato leaves treated with NaCl (140 and 210 mM) was accompanied by a reduction in chlorophyll content, reduction of photosynthetic efficiency (Fv/Fm), and an elevation of H_2_O_2_ level (Chen et al., 2012) consistent with the down regulation of genes involved in photosynthesis observed in this study. Specifically, we observed down regulation of the light‐harvesting in photosystem I and II; NDHK, PHOTOSYSTEM II REACTION CENTER PROTEIN G (PSBG; itf00g02070, LFC = −2.9218, *p*‐value .003), PHOTOSYSTEM I LIGHT HARVESTING COMPLEX GENE 3 (LHCA3; itf02g14020, 24 h, LFC = −2.4465, *p*‐value 2.21E−10), PHOTOSYSTEM II REACTION CENTER PROTEIN D (PSBD; itf02g22380, LFC = −2.3226, *p*‐value, 6.34E−5) and LIGHT HARVESTING COMPLEX PHOTOSYSTEM II (LHCB; itf12g10840, 24 h, LFC = −2.2464, *p*‐value 5.24E−5).

Auxin is involved in the spatial regulation of plant growth and development and has been implicated in regulating key plant processes including root and lateral root development, root gravitropism, root hair development, vascular patterning and development, seed germination, apical hook formation, leaf morphogenesis, phyllotactic patterning, female gametophyte development and embryo development. Through specific signaling pathways Auxin plays a role in adaptive responses to various biotic and abiotic stresses (Semeradova et al., 2020; Swarup & Bhosale, 2019). Approximately 85 genes encoding Auxin and Auxin‐responsive proteins such as the SAUR‐like Auxin‐responsive protein family were down regulated following salt stress (Table S8 and S9). Multiple SAUR Auxin‐responsive genes were up regulated by salt stress including SAUR9 (itf09g00770, 24 h, log2fc = −3.8313, *p‐*value 4.57E−5), SAUR10 (itf12g02790, 24 h, log2fc = −3.1732, *p‐*value 2.57E−7), and SAUR3 (itf09g24220, 48 h, log2fc = −2.8417, *p‐*value .003; Table S9). Hormones including Auxin (Hagen & Guilfoyle, 2002; van Mourik et al., 2017) as well as high‐temperature (Franklin et al., 2011), drought and high salt conditions (Guo et al., 2018; Wu et al., 2012) can modulate SAUR gene expression and multiple SAUR‐like genes which were differentially regulated under both heat and salt stress (Table S9).

Sugar Will Eventually be Exported Transporters (SWEET) in plants are associated with adaption to adverse environmental conditions including abiotic stress tolerance, that is, drought, cold, heat, and salt stress (Chandran, 2015; Chen et al., 2010; Durand et al., 2016; Gautam et al., 2022; Kafle et al., 2019; Lu et al., 2019; Wei et al., 2019; Zhou, Ma, et al., 2018). Significant expression changes of the SWEET gene family occurred following single and multiple abiotic stress treatments, including drought, heat, heat combined with drought, and salt stress, and were more up regulated in response to drought and salt stresses in wheat (Qin, Jiang, et al., 2020) and rice (Mathan et al., 2021). Transcript levels of the Sweet15 gene was reported to be up regulated up to 64‐fold higher than the control in Arabidopsis during salt stress (van Zelm et al., 2020; Zhao et al., 2019). Similarly, the expression of MaSWEETs was induced by cold, salt, and osmotic stresses in banana (Miao et al., 2017). In Beauregard, a SWEET gene (itf08g07850, 24 h, log2fc = 4.0582, *p‐*value 1.05E−9; Table S5) was up regulated following salt stress.

### 3.4 Clustering and co‐expression network analyses

**FIGURE 5:**
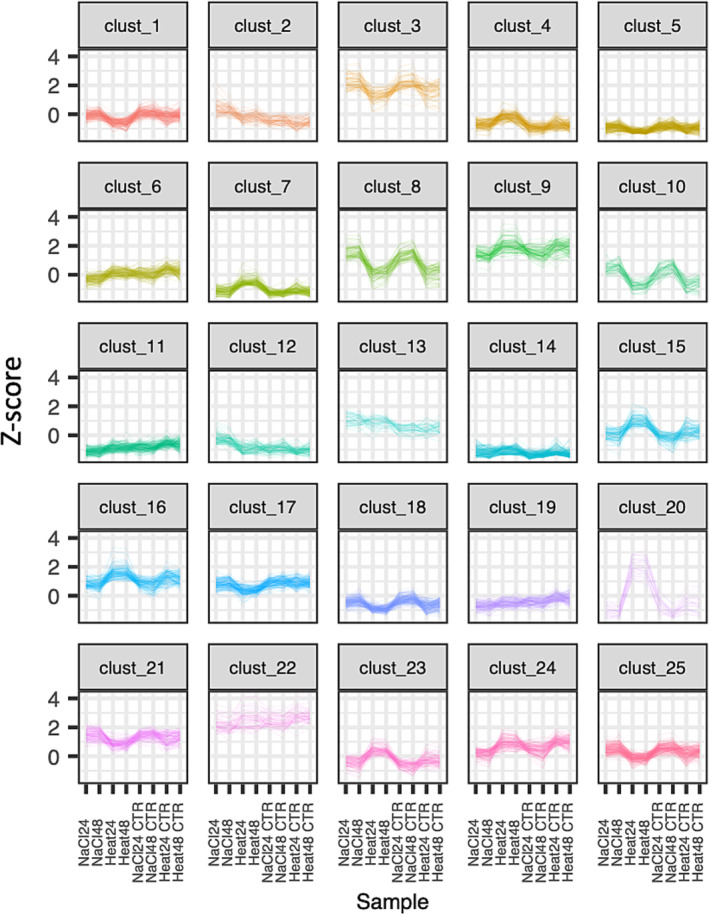
Clustering of gene expression profiles of 3238 differentially expressed genes by K‐means clustering method to identify co‐regulated genes following heat and salt stress treatment of Beauregard leaf tissue.

Clustering of gene expression patterns is useful in understanding and annotating gene function as “guilt‐by‐association” can be used to infer gene function. To associate patterns of expression with a stress response, we used the supervised K‐means approach and clustered 3238 of the 3346 DEGs (after filtering overlapping genes) into 25 clusters (Figure 5 and Table S12). While most clusters showed no specific pattern associated with stress, clust 15 genes (*n* = 110) and clust 20 (*n* = 27) were associated with up regulation during heat stress and enriched for genes associated with heat responses as described above (Table S13). Genes that were enriched for clust 15 and 20 included multiple HSPs and HSFs, most of which were up regulated in heat‐treated samples but down regulated in salt stress.

#### 3.4.1 Co‐expression network analysis reveals modules of co‐regulated genes

Expression abundances (FPKMs) from the heat and salt stress samples were combined with simulated drought dataset that simulated drought (Lau et al., 2018) to identify genes co‐expressed and potentially co‐regulated in response to all three stresses. Weighted gene co‐expression network analyses identified 18 modules with gene membership ranging from 34 (dark olive green) to 991 genes (salmon) (Figure S1) that were enriched in different biological functions (Table S14). The blue module was enriched for “response to water deprivation” (GO:0009414, *p*‐value; .00012), “response to abscisic acid” (GO:0009737, *p*‐value; .00119), “nitrate transport” (GO:0015706, *p*‐value; 2.80E−5), and “response to nitrate” (GO:0010167, *p*‐value; .00011), among others, suggesting it was associated with drought. Under low N and drought conditions, stomata remain closed; however, combined drought and high N conditions can stimulate stomata to remain open, leading to higher transpiration rates and resulting in greater water use (Shi et al., 2014) thus delaying drought effects (Ren et al., 2015). The HIGH‐AFFINITY NITRATE TRANSPORTER 2.7 gene (ATNRT2.7, NRT2; itf03g00630) was up regulated under both drought (log2fc = 4.6632, *p‐*value 2.49E−37; Table S15) and salt stress conditions (log2fc = 3.1758, *p‐*value 1.20E−11) consistent with previous reports of synergetic crosstalk between N and water transport during water stress (Ding et al., 2018). *AtNRT2.7* has been identified as one of the seven high‐affinity nitrate transporters in Arabidopsis involved not only in uptake and transport of NO_3_ and its translocation, (Huang et al., 1999; Orsel et al., 2006; Wang et al., 2012) but also in transporting other biological compounds including ABA. Araus et al. (2020) showed that changes in NO_3_^−^ availability may lead to changes in the expression of drought‐responsive genes. Three genes in the blue drought‐associated module encoded TFs associated with drought stress responses including zf‐C_2_H_2_ (Cystein2/Histidine; itf11g04060) and HB‐ > HB‐HD‐ZIP (itf12g07740, itf14g03710) consistent with reports from Arisha, Ahmad, et al. (2020) who found several gene families including C_2_H_2_ and HD‐ZIP transcription factor family members responding to drought stress in sweetpotato. Transformation of Arabidopsis with *IbZFP, a* C_2_H_2_‐type zinc finger protein from sweetpotato, improved plant drought resistance (Wang et al., 2016) leading to less leaf water loss, lower content of ROS, higher leaf water content, and higher antioxidant enzyme activities after drought treatment of the transgenic plant.

Various TF pathways operate in ABA‐dependent and ‐independent signaling pathways mediating transcriptional regulation of gene expression leading to the expression of early response transcriptional activators and activation of downstream stress tolerance effector genes. The blue drought module contained 63 genes significantly enriched for the GO term “response to abscisic acid” (GO:0009737, *p‐*value .00119, Table S14) with 15 encoding members of multiple TF families. Extensive studies have shown that, in addition to their functions in plant growth and development, NAC transcription factors play a key role in abiotic stress responses (Shao et al., 2015). Transgenic Arabidopsis plants overexpressing either of these paralogs, ANAC019, ANAC055, or ANAC072 (itf05g25290), show significantly increased drought tolerance (Tran et al., 2004). In our samples, a NAC gene (itf05g25290) was present in the drought‐associated module (blue) and was differentially up regulated during drought and salt stress (itf05g25290, salt; 48 h, LFC = 2.3674, *p*‐value 2.81E−7; drought; 48 h, LFC = 7.0490, *p*‐value 1.83E−53; Table S3). In Arabidopsis, ARABIDOPSIS THALIANA HOMEOBOX 7 (ATHB‐7) and AtHB12 genes are strongly up regulated after osmotic or drought stresses in young plants upon ABA or NaCl treatment (Lee & Chun, 1998; Olsson et al., 2004; Söderman et al., 1996). Furthermore, ectopic expression of AtHB7 confers drought tolerance to transgenic tomato (Mishra et al., 2012). The homeobox gene itf05g19720 was dup regulated in response to salt (LFC = 3.7014, *p‐*value 1.84E−20) and drought (LFC = 8.9468, *p*‐value 1.20E−86) at 48‐h time point in our study.

#### 3.4.2 Key hub genes associated with drought sensitivity in sweetpotato

**FIGURE 6:**
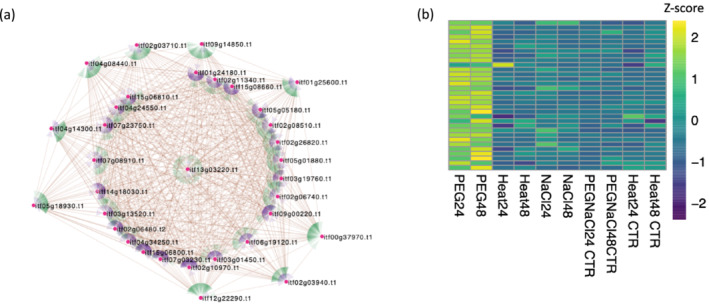
Weighted gene co‐expression network analysis (WGCNA). (a) Co‐expression network including 32 highly connected nodes (genes, magenta) from the drought (blue) module. Targeted and source nodes are indicated by green and purple colors, respectively. (b) Heatmap displaying expression (Z‐score) of (blue) drought network genes from heat, salt (NaCl) and drought (PEG) treated Beauregard, leaf tissue.

Within the drought‐associated (blue) module, osmotin (itf13g03220, LFC = 5.9807, *p*‐value 8.67E−108; 48 h) was the highest connected (*ktotal* and *kwithin*; Figure 6 and Table S15). Osmotin, a multifunctional stress‐responsive protein, belongs to the pathogenesis‐related 5 (PR‐5) defense‐related protein family and imparts drought, salt, and cold tolerance (Goel et al., 2010; Husaini & Abdin, 2008; Le et al., 2018; Patade et al., 2013; Wan et al., 2017; Weber et al., 2014; Zhu et al., 1995). During stress conditions, the accumulation of the osmolyte proline which quenches ROS and free radicals is facilitated by osmotin. Furthermore, osmotin from the resurrection plant *Tripogon loliiformis* confers numerous simultaneous abiotic stresses tolerance (cold, drought, and salinity) in transgenic rice (Le et al., 2018; Mandal et al., 2018). Overexpression of the osmotin gene in early stages of osmotic stresses (cold, drought, and salinity) by a 1,000‐fold conferred resistance against abiotic stress in transgenic *Nicotiana tabacum*, *Oryza sativa*, and sesame (Chowdhury et al., 2017) while in potato, osmotin over expression was found to cause delays in the development of late blight disease symptoms (Liu et al., 1994). As a result, osmotin has been proposed as a high‐value gene for developing multiple stress‐tolerant biofortified crops (Husaini, 2022).

Among the terms enriched for the blue, drought module, were genes in response to “extracellular region” (GO:0005576, *p*‐value; 4.50E−14). Furthermore, two expansin A15 genes found in our network analysis were up regulated in the drought‐stressed samples; itf02g03710 (LFC = 9.3730, *p*‐value 5.14E−97) and itf02g03940 (LFC = 9.7680, *p*‐value 3.87E−79) at 48 h. Expansins are induced by phytohormones, biotic and abiotic stresses such as heat, drought, salt and heavy metals and are involved in cell expansion and cell wall changes (Cosgrove, 2000; Ding et al., 2016; Le Gall et al., 2015; Lu et al., 2016; Xu et al., 2014). It has been shown that the overexpression of expansin genes in Arabidopsis enhances plant tolerance to drought stress, salt stress (Dai et al., 2012), tolerance to drought in transgenic tobacco, (Li et al., 2011), high salt in wheat (Han et al., 2012), and rice (Jadamba et al., 2020), and drought and heat tolerance in potato (Chen et al., 2019). Interestingly, besides drought and salt having common response patterns, osmotin and expansin A15 genes were not differentially expressed in salt stress samples, which may suggest the presence of specifically affected biochemical pathways by drought.

## 4 CONCLUSIONS

Characterization of heat, salt, and drought‐responsive gene expression in an OFSP cultivar identified stress specific as well as shared responses to the stress treatments. A key impact of abiotic stress was evident from the down regulation of photosynthesis, arrest of the cell cycle, and activation of a canonical heat shock response. We identified crosstalk signaling between genes, TFs and phytohormones. Collectively, this study illustrated the genes responding to heat, salt and drought and serve as a potential application of TF genes for stress tolerance improvement and the engineering of stress resistant sweetpotato varieties and other crops.

## AUTHOR CONTRIBUTIONS

DCG and AK designed and executed the experiments and isolated RNA. SW and ZF sequenced the RNA for gene expression profiling. MK performed RNAseq bioinformatics analysis guided by JW, JH and CRB. Data visualization and manuscript writing was performed by MK and CRB. All authors read and approved the manuscript.

## CONFLICT OF INTEREST STATEMENT

The authors declare that the research was conducted in the absence of any commercial or financial relationships that could be construed as a potential conflict of interest.

## PEER REVIEW

The peer review history for this article is available in the Supporting Information for this article.

## Supporting information


**Figure S1.** WGCNA clustering dendrogram of genes obtained by hierarchical clustering of adjacency‐based dissimilarity of 14,138 genes from the heat, salt and drought treated Beauregard leaf tissue. Co‐expression modules were identified via the Dynamic Tree Cut method; the merged dynamic indicates modules divided according to similarity of the module (with assigned module colors). Analysis was carried out according to the merged modules. Vertical distance in tree diagram represents distance between two nodes (between genes).

**Table S1.** Summary of RNA‐Seq libraries. Number of reads, reads passing filter after cleaning and alignment metrics to the reference genome *Ipomoea trifida.*

**Table S2.** FPKMs Pearson's Correlation Coefficients between and among biological replicates derived from RNAseq libraries of heat (40 °C) stress, salt (NaCl) stress and drought (25% PEG) stress treated Beauregard plants and control samples.

**Table S3.** Differential expression of all significant (*p*‐value < .05) up regulated (LFC > 2.0) and down regulated (LFC < − 2.0) genes at 24 hr and 48 hr time points following heat (40 °C) stress, salt (NaCl) stress and drought (25% PEG) stress treatments on Beauregard sweetpotato cultivar.

**Table S4.** Unique differentially up regulated (LFC > 2.0; *p*‐value < .05) genes in sweetpotato leaf tissues following heat (40 °C) stress treatment.

**Table S5.** Unique differentially up regulated (LFC > 2.0; *p*‐value < .05) genes in sweetpotato leaf tissues following salt (NaCl) stress treatment.

**Table S6.** Common differentially up regulated genes (LFC > − 2.0; *p*‐value < .05) in leaf tissues following heat (40 °C) stress and salt (NaCl) stress treatments on Beauregard sweetpotato plants.

**Table S7.** Unique differentially down regulated genes (LFC < − 2.0; *p*‐value < .05) in leaf tissues following heat (40 °C) stress treatment on Beauregard plants.

**Table S8.** Unique differentially down regulated genes (LFC < − 2.0; *p*‐value < .05) in leaf tissues following salt (NaCl) stress treatment on Beauregard plants.

**Table S9.** Common differentially down regulated genes (LFC > − 2.0; *p*‐value < .05) in leaf tissues following heat (40 °C) stress and salt (NaCl) stress treatment on Beauregard plants.

**Table S10.** Gene ontology (GO) terms enriched for the up and down differentially regulated genes following heat (40 °C) stress and salt (NaCl) stress treatments on Beauregard sweetpotato leaf tissue at 24 hr and 48 hr.

**Table S11.** Expression (gene counts as log CPM) of heat shock‐related genes in heat (40 °C) stress and salt (NaCl) treated Beauregard sweetpotato leaf tissue.

**Table S12.** Differentially regulated genes grouped into 25 clusters using K‐means based on the expression patterns (Z‐score) in the treated and control samples with transcription factor information.

**Table S13.** Gene ontology (GO) term enrichment of genes in K‐means clusters.

**Table S14.** Gene ontology enrichment of Weighted Gene Co‐expression Network Analysis (WGCNA) modules.

**Table S15.** Intra (*kwithin*) and inter (*ktotal*) modular connectivity measuring of genes in the Weighted Gene Co‐expression Network Analysis.

**Data S1.** Supporting Information.

## Data Availability

Raw sequence reads have been deposited in the National Center for Biotechnology Information Sequence Read Archive BioProject PRJNA834099 and PRJNA834095.
