# Supplementary material for: Identification of genes associated with abiotic stress tolerance in sweetpotato using weighted gene co‐expression network analysis
Source: Plant Direct. 2023 Oct 3;7(10):e532. doi: 10.1002/pld3.532 (PMC10546384; doi:10.1002/pld3.532)
Supplement: Supplementary file 1 — Figure S1. WGCNA clustering dendrogram of genes obtained by hierarchical clustering of adjacency‐based dissimilarity of 14,138 genes from the heat, salt and drought treated Beauregard leaf tissue. Co‐expression modules were identified via the Dynamic Tree Cut method; the merged dynamic indicates modules divided according to similarity of the module (with assigned module colors). Analysis was carried out according to the merged modules. Vertical distance in tree diagram represents distance between two nodes (between genes). [file PLD3-7-e532-s015.docx]

**Supplementary Materials**

**Supplementary Figures**

**Figure S1**. WGCNA clustering dendrogram of genes obtained by hierarchical clustering of adjacency-based dissimilarity of 14,138 genes from the heat, salt and drought treated Beauregard leaf tissue. Co-expression modules were identified *via* the Dynamic Tree Cut method; the merged dynamic indicates modules divided according to similarity of the module (with assigned module colors). Analysis was carried out according to the merged modules. Vertical distance in tree diagram represents distance between two nodes (between genes)

**
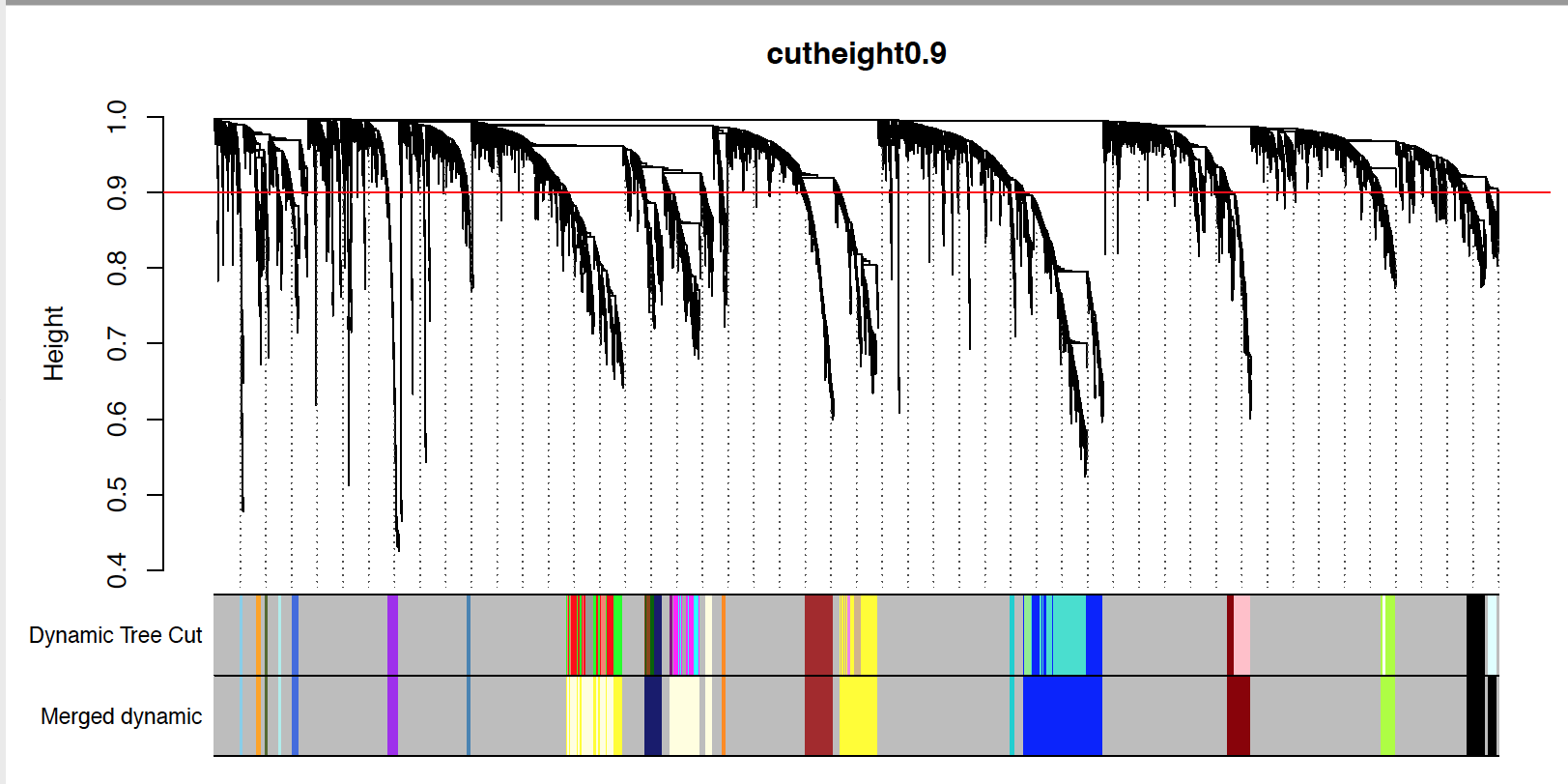
**
