# Supplementary material for: Identification of genes associated with abiotic stress tolerance in sweetpotato using weighted gene co‐expression network analysis
Source: Plant Direct. 2023 Oct 3;7(10):e532. doi: 10.1002/pld3.532 (PMC10546384; doi:10.1002/pld3.532)
Supplement: Supplementary file 17 — Data S1. Supporting Information. [file PLD3-7-e532-s014.docx]

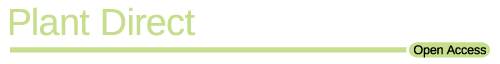


DECISION LETTER – Round 1

| April 22, 2023  Dr. Mercy Nzilani Kitavi Michigan State University Research Technology Support Facility 612 Wilson Road S-20 East Lansing, Michigan 48824   RE: Identification of genes associated with abiotic stress tolerance in sweetpotato using weighted gene co-expression network analysis  Dear Dr. Kitavi:  Thank you for submitting to Plant Direct. All required reviews have been returned and we have now finished our evaluation of your manuscript. In light of the reviewers' and editor's comments, further revisions are needed before the paper can be accepted for publication in Plant Direct.  Please view the editors' and reviewers' comments below and use their suggestions as a guide while you work on your revision.  When uploading the revised version of this article, please be sure to include the following:  -A word document that contains your response to the reviewers. You should respond to each reviewer comment and note the changes made to the manuscript. If you do not agree with a reviewer's comment and choose not to make a suggested revision, please explain why. Please try to provide as complete an answer as possible to each reviewer's criticisms. -A tracked changes document with each change highlighted - A clean version of the latest version of the manuscript  The authors should address the referees comments  To upload your revision, please click the link below. https://plantdirect.msubmit.net/cgi-bin/main.plex?el=A4Lr2ZK4A5nto6I4A9ftdkyBq5f1wiE3peeXvuhFlgZ   In order to provide as timely a service as possible, we ask that your revision is resubmitted within three months after receipt of this request. If an extension is needed, please send a request, along with a brief explanation, to the editorial office at plantdirect@wiley.com .  Please note that, in addition to publishing reviewer comments, the author's responses to review comments will also be published alongside the final version of the paper. If you would not like the author's responses to be published, please contact the editorial office at plantdirect@wiley.com .   Thank you very much for giving us an opportunity to review your work. I look forward to receiving the next version.   Sincerely,  Ana Fortes  Ana Fortes  Editor, Plant Direct   ---------------------------------------------------------------------------- Editor comments: The authors should address the referees comments  ---------------------------------------------------------------------------- Reviewer comments: Reviewer #1:  All comments are in the attached file. Materials and Methods part the following comments: What plant part was used? How was the environment sterilized, mentioning the name of the device and the manufacturer? In which package was the cultivation, mentioning its size? What is the point of changing the temperature, now we usually use 27 degrees Celsius How much is the addition of this compound?   Reviewer #2:  Kitavi et al, present transcriptomic analysis of sweet potatoes under salt and heat stress. The study in itself provides no new insights into how these abiotic stress responses affect the gene expression profile, in fact is an extension of earlier study by the group under drought stress. However, the authors in this study present a transcriptomic profile of the sweet potatoes leveraging the newly available genome, providing the knowledge about the genes that are perturbed under two different kinds of environmental stresses, in this case salt and heat stress.  The manuscript is well written, clear and easy to understand. The dataset might be valuable for the plant scientific community working on the stress response. However, before the MS can be accepted, I have few questions:  Major:  The authors should clarify what were their controls? Meaning whether they left the plants without treatment and collected at 24 h and 48h or the same controls were used for both treatment.  Fig 1A- the PCA plot is not very clear. The authors argue that the largest variation is between stress and control but does not look like that, specially salt stress. Can the authors explain this?  The salt stress treated leaves at 48 HAS showed relatively less DEGs compared to 24h. Can the authors explain the reason behind this?  Fig 2: it would be interesting to look at upregulated genes due to the salt and heat stress. Maybe a supple file would be enough. The order of fig 2 also needs to be considered. It should possibly come after 3 and 4.  Minor:  Line 64: What does "address vitamin A" mean? Is it a deficiency?  Line 74-75- abiotic and biotic stress? Line 107- suggestive? Fig 3B: Heatmap labels should indicate that the plot shows the average of three reps if that's the case  GO plots in figure 2, 3 and 4 should be consistent. Either all BP, CC, and MF should be plotted for all figures and only one BP as in Figure 2.  For better readability, the supplementary tables at the end should be provided in the excel file.   Reviewer #3:  This paper deserves to be published and represents a nice study of abiotic stress(es)-induced transcriptional changes in sweetpotato leaves. Some critical points need to be addressed before the manuscript is ready for publication. Please see the attached file. |
| --- |

| **Attachment:** | [Reviewer 1 Review Attachment 1 - 04-22-2023 05:31:29](https://plantdirect.msubmit.net/pd_files/2023/01/28/00001310/00/1_reviewer_attachment_1_1679017378.pdf) |
| --- | --- |
| **Attachment:** | [Reviewer 3 Review Attachment 1 - 04-22-2023 05:31:29](https://plantdirect.msubmit.net/pd_files/2023/01/28/00001310/00/3_reviewer_attachment_1_1682115272_convrt.pdf) |

DECISION LETTER- Round 2

August 31, 2023
Dr. Mercy Nzilani Kitavi
Michigan State University
Research Technology Support Facility
612 Wilson Road S-20
East Lansing, Michigan 48824


MSID: 2023-01127R1
MS TITLE: Identification of genes associated with abiotic stress tolerance in sweetpotato using weighted gene co-expression network analysis

Dear Dr. Mercy Kitavi:

I am pleased to inform you that your manuscript "Identification of genes associated with abiotic stress tolerance in sweetpotato using weighted gene co-expression network analysis" has been accepted for publication in Plant Direct.

Your article will appear online in the next available issue of Plant Direct. To ensure your article gets published as quickly as possible, please pay attention to the steps detailed below. We have found that most of the delays happen at this stage, especially at the payment stage, so please respond as quickly as possible when prompted.

License Agreement: Once your article has been accepted it will move to Production and undergo admin and file checking - you may receive an email with any queries we have at this stage. When all required items are received by the publisher and queries resolved, the corresponding author will receive an email from Wiley's Author Services system which will ask them to log on at https://authorservices.wiley.com/bauthor and will present them with the appropriate license for completion. Your article cannot be published until both the signed license agreement and payment of the article fee have been received.

Payment of the Open Access Article Publication Fee: All articles published in Plant Direct are fully open access: immediately and freely available to read, download and share. Plant Direct charges a publication fee to cover the publication costs. The corresponding author for this manuscript should have already received a quote from the payments team (cs-openaccess@wiley.com) with the estimated article publication fee; please email cs-openaccess@wiley.com if this has not been received. The corresponding author should log on to the Wiley Author Services site, where the publication fee can be paid by credit card or an invoice. Pro Forma can also be requested. Payment of the publication charge must be received before the article will be published online.

Proofs: You will have the opportunity to look over your paper once more when you receive the author proofs for your article. The proofs will be with you in approximately two weeks. Please note that, in addition to publishing reviewer comments, the author's responses to review comments will also be published alongside the final version of the paper. If you would not like the author's responses to be published, please contact the editorial office at plantdirect@wiley.com .

Promotion of your article: You can help your research get the attention it deserves! Check out Wiley's free Promotion Guide for best-practice recommendations for promoting your work at www.wileyauthors.com/eeo/guide. And learn more about Wiley Editing Services which offers professional video, design, and writing services to create shareable video abstracts, infographics, conference posters, lay summaries, and research news stories for your research at www.wileyauthors.com/eeo/promotion.

Thank you again for your contribution to Plant Direct. If you have any questions, feel free to contact the editorial office at plantdirect@wiley.com .

Sincerely,

Ana Fortes

Ana Fortes

Editor, Plant Direct


---------------------------------------------------------------------------- Editor comments


---------------------------------------------------------------------------- Reviewer comments:

AUTHORS’ RESPONSE

Dear Dr. Fortes,

We appreciate the comments from the reveiwers and have revised our manuscript to incorporate their suggestions. We have uploaded our revised manuscript, a marked up version to highlight the changes and a point-by-point response to the reviewers comments. We hope our manuscript is now suitable for publication in Plant Direct.

Sincerely

Mercy Kitavi

Reviewer #1:

All comments are in the attached file.

Author Response: A number of the comments in the marked up PDF from Reviewer 1 are on grammar and corrected these as appropriate throughout the manuscript. We do want to highlight two issues with this reviewer’s suggestion on grammar (see below). For all other specific comments by Reviewer #1, we provide a point-by-point response below.

Grammar:

Sweetpotato Is One Word: This work was done in collaboration with and funded by a grant awarded to North Carolina State University. In 1989, the [National Sweetpotato Collaborators Group](https://sweetpotatocollaborators.wordpress.ncsu.edu/) changed their spelling to one word and advocated for those in academia to do the same. Thus, we have adopted use of sweetpotato in this manuscript.

Upregulation, Downregulation, Upregulated and Downregulated

**Corrected to:**up regulation, down regulation, Up regulated and Down regulated according to the Oxford Dictionary of Biochemistry and Molecular Biology (2 ed.)

Other Comments from Reviewer #1:

c11: It is suggested to increase the information on the available studies on standardized vitamins, with support by numbers

Author Response: Additional text added.

c12: I suggest increasing information about the available items and their spread around the world, with support by numbers

Author Response: Additional text added.

c17: Cite some biotic and abiotic stresses

Author Response: We have edited the text to address this comment.

c18: Cite some plants to illustrate environmental stresses

Author Response: We have listed plant species to which the references have been cited

c19: On what basis were the references placed, is it year or alphabetical o rwhat?

Author Response: The references have been rearranged based on alphabetical order

c20: typographical error

Author Response: Corrected

c27-33: Experimental details requested

Author Response: The experimental design and sampling is now well described in the revised Materials and Methods.

c35-39:

Author Response: These are the correct syntax for use of this software.

c45-46, 88, 91: Add recent references

Author Response: Additional references added

c47: topGO

Author Response: The topGO package is designed to facilitate semi-automated enrichment analysis software for Gene Ontology (GO) terms. It is written correctly in the text.

c99: Add recent references

Author Response: Recent refences have been added.

c99: Add some details about cell growth with references mentioned

Author Response: Additional details have been added.

c105: Mention some details

Author Response: More details of abiotic stresses affected by SWEET genes have been mentioned.

C123: Needs more results

Author Response: We added a limited amount of additional text as the Conclusion section is meant to be a short summary of the manuscript.

Materials and Methods part the following comments:
What plant part was used?

Author Response: The experimental design and sampling is now well described in the revised Materials and Methods.

How was the environment sterilized, mentioning the name of the device and the manufacturer?

Author Response: Details on the environment and sterilization methods are provided in the revised Materials and Methods.

In which package was the cultivation, mentioning its size?

Author Response: Details on the cultivation methods are provided in the revised Materials and Methods.

What is the point of changing the temperature, now we usually use 27 degrees Celsius

Author Response: The temperatures used were selected based on literature reports and experience in growing sweetpotato.

How much is the addition of this compound?
Author Response: Details on the concentration of calcium pantothenate are provided in the text

Reviewer #2:

Kitavi et al, present transcriptomic analysis of sweet potatoes under salt and heat stress. The study in itself provides no new insights into how these abiotic stress responses affect the gene expression profile, in fact is an extension of earlier study by the group under drought stress. However, the authors in this study present a transcriptomic profile of the sweet potatoes leveraging the newly available genome, providing the knowledge about the genes that are perturbed under two different kinds of environmental stresses, in this case salt and heat stress.

The manuscript is well written, clear and easy to understand. The dataset might be valuable for the plant scientific community working on the stress response. However, before the MS can be accepted, I have few questions: 

Major:

The authors should clarify what were their controls? Meaning whether they left the plants without treatment and collected at 24 h and 48h or the same controls were used for both treatment.

Author Response: The experimental design and sampling is described in more detail in the revised Materials and Methods.

Fig 1A- the PCA plot is not very clear. The authors argue that the largest variation is between stress and control but does not look like that, specially salt stress. Can the authors explain this?

Author Response: In Figure 1a, the PEG treated samples account for most of the variance, thus, distorting the differences between salt/heat stress and their appropriate controls. Salt stress and control seem to have minimal separation using PC1 and PC2 but it does not mean that no genes differ between salt stress and their control samples. It is just that they don’t explain most of the variance captured by PC1 and PC2, they may be distinguishable if we explore other PC axis.

The salt stress treated leaves at 48 HAS showed relatively less DEGs compared to 24h. Can the authors explain the reason behind this?

Author Response: It could be that by 48 HAS, adaptation to the saline conditions has occurred. An explanation has been added in the text

Fig 2: it would be interesting to look at upregulated genes due to the salt and heat stress. Maybe a supplementary file would be enough. The order of fig 2 also needs to be considered. It should possibly come after 3 and 4. 

Author Response: Supplemental Tables 4 and 5 list genes that are up regulated by heat and salt stress. Supplemental Table 6 shows genes that were up regulated by both stress treatments

Minor:

Line 64: What does "address vitamin A" mean? Is it a deficiency?

Author Response: We have corrected this text to include deficiency

Line 74-75- abiotic and biotic stress?

Author Response: We have edited the text to address this comment.

Line 107- suggestive?

Author Response: Corrected

Fig 3B: Heatmap labels should indicate that the plot shows the average of three reps if that's the case

Author Response: Text added on number of replicates.

GO plots in figure 2, 3 and 4 should be consistent. Either all BP, CC, and MF should be plotted for all figures and only one BP as in Figure 2.

Author Response: All Figures; 2, 3 and 4 show terms enriched in the BP category. Figure 2 shows terms affected by DEGs commonly downregulated by heat and salt stress, the figure legend has been amended to reflect that all terms are BP. Due to the low number of DEGs (52) commonly up regulated by the two stress conditions, we only report on the BP associations for down regulated DEGs.

Figure 3 has been redone to include terms affected by both up regulated and down regulated.

For better readability, the supplementary tables at the end should be provided in the excel file.

Author Response: This comment is unclear as the supplementary tables were provided as an excel file.

Reviewer #3:

This paper deserves to be published and represents a nice study of abiotic stress(es)-induced transcriptional changes in sweetpotato leaves.
Some critical points need to be addressed before the manuscript is ready for publication.

Comments:

• It is not clear if the three stress treatments were conducted at the same time in one experiment or in three independent experiments at different times (this would also explain why the transcription profile of control plants is so different in the PCA figure). From the PCA figure is clear that control plants of heat and salt are very different. As much (or more in the case of salt) as their respective stress treatments.

Author Response: The experimental design and sampling is now well described in the revised Materials and Methods. The salt and drought simulation experiment were done at a different time than the heat stress.

• Considering that Figure 1d is based on DEGs only, the heatmap should show hierarchical clustering for the samples as well, not only for the genes (after adding PEG treated samples too)

Author Response: The figure has been updated.

• In Figure 2, there are three GO terms enriched categories of DEGs induced by heat stress and relative to biosynthetic processes: unsaturated fatty acid, phosphatidylglycerol, and pentacyclic triterpenoid. There is a vast literature on lipid membrane remodeling under heat and drought stress, as well as terpene synthesis in response to stress. These three categories are pointing into that direction and should be discussed in the text.

Author Response: The text has been updated with a discussion of lipids and stress responses.

• In L282-284, the decrease of chlorophyll content is described in the context of a possible decreased stomatal closure. This is a bit speculative and could be tested by checking if transcripts associated with photosynthesis decreased (e.g. Rubisco) or with photorespiration increased (e.g. glycolate oxidase).

Author Response: The text has been updated with a discussion of genes associated with photosynthesis.

• Figure 3a and Figure 4. I find that there is a discrepancy in the way these two figures are presented for heat and salt stress. Categories are not used for salt stress, why? Also, in Figure 4 I understand what categories are up- and down-regulated. Why in Figure 3a only the upregulated are represented?

Author Response: The discrepancy in Figures 3a and 4 has been addressed. Figure 3a has been redone to show Biological process terms affected by both up regulated and down regulated DEGs
